# Determinants of intentions to quit smoking among adult smokers in Bangladesh: findings from the International Tobacco Control (ITC) Bangladesh wave 2 survey

**DOI:** 10.1186/s41256-016-0012-9

**Published:** 2016-08-17

**Authors:** Pete Driezen, Abu S. Abdullah, Anne C. K. Quah, Nigar Nargis, Geoffrey T. Fong

**Affiliations:** 1 Department of Psychology, University of Waterloo, Waterloo, ON Canada; 2 Global Health Program, Duke Kunshan University, Kunshan, Jiangsu Province 215347 China; 3 Duke Global Health Institute, Duke University, Durham, NC 27710 USA; 4 Department of General Internal Medicine, Boston University School of Medicine, Boston Medical Center, 801 Massachusetts Avenue, Crosstown Center (2nd Floor), Boston, MA 02118 USA; 5 Department of Economics, University of Dhaka, Dhaka, Bangladesh; 6 American Cancer Society, Washington DC, USA; 7 Ontario Institute for Cancer Research, Toronto, ON Canada

**Keywords:** Smoking, Intention to quit, Bangladesh, Determinants

## Abstract

**Background:**

With about 22 million adult smokers, Bangladesh needs strong measures that would promote smoking cessation. Using data from Wave 2 of the International Tobacco Control (ITC) Survey, this study examined the factors associated with intention to quit smoking among Bangladeshi smokers.

**Methods:**

Data from Wave 2 of the International Tobacco Control (ITC) Survey in Bangladesh, a face to face survey of adult smokers, were analysed. In the ITC survey, households were sampled using a stratified multistage design and interviewed using a structured questionnaire.

**Results:**

Of the respondents (*N* = 2982), most were male (96 %), married (80 %), and Muslim (83 %); 33 % were illiterate and 54 % were aged below 40. Almost two-thirds were from areas outside Dhaka, 78 % smoked cigarettes exclusively; and 36 % had an intention to quit smoking in the future. This study identified several predictors, comparable to other international studies, of intention to quit smoking: area of residence, number of cigarettes smoked daily, previous quit attempt, visiting a doctor in the past, having child aged 5 or below at home, perceived benefit from quitting, being worried about own health, knowledge of SHS, not enjoying smoking and workplace smoking policy.

**Conclusions:**

These findings suggest that the prevalence of intention to quit smoking is lower among Bangladeshi smokers than those among smokers in developed countries. However, the factors relating to quit intentions among Bangladeshi smokers are comparable to those found in Western countries. Population based tobacco control programs and policies should consider these predictors in the design of interventions to increase quitting among smokers in Bangladesh.

## Background

Tobacco smoking, including second hand smoke, is one of the leading risk factors for global disease burden accounting for 6.3 % of global disability adjusted life years (DALYS) [1]. Annual deaths attributed to tobacco smoking will rise from approximately 5 million in 2010 to more than 10 million in the next few decades if the current patterns of smoking and quitting do not change [2, 3]. The 2013 World Health Assembly called on governments to reduce the prevalence of smoking by a third by 2025, which would prevent more than 200 million deaths from tobacco during the remainder of the century [2]. Promotion of smoking cessation has been proposed as one of the primary areas of focus for tobacco control in developing countries [4].

Quitting smoking is beneficial and increases life expectancy among smokers [1], regardless of the timing of cessation. However, giving up smoking is not a single act. Rather, smoking cessation is a process that progresses through several stages described by Prochaska and DiClemente in their transtheoretical stages of change model [5]. As people’s readiness to change shifts when they move through the various stages, interventions must be tailored to target the specific stage [6, 7].

Previous research in different countries has identified several factors associated with intentions to quit, including socioeconomic factors [8–11], positive attitude towards quitting [12–14], perceiving more behavioral control over quitting [13, 14] perceiving quitting as a priority [14], health concerns [12, 15], having received medical advice to quit smoking [15], perceived social pressure [16, 17], expected social support for quitting [15, 17], higher self-efficacy [16], having confidence to quit [12], being older [12, 18], consuming fewer cigarettes [13, 19, 20], lower nicotine dependence level [21], and past quitting attempt [12, 13, 16, 20].

A study of Korean adults found that sociodemographic factors, smoking-related beliefs, and smoking restrictions at home were associated with intention to quit smoking [22]. In another study of adult Chinese smokers, many of these same factors (i.e., past quit attempts, duration of past quit attempts, nicotine dependence level, outcome expectancy and opinion of smoking) were associated with intentions to quit smoking, although demographic factors were not [23].

Bangladesh is one of the top ten countries in the world with high current smoking prevalence of 44.7 % among men [24]. This country is distinguished as the first signatory of the World Health Organization (WHO) Framework Convention on Tobacco Control (WHO FCTC) which was ratified on 10 May 2004. The ratification was made concrete with the passage of the Tobacco Control Act (TCA) on 15 March 2005. To our knowledge, no population based studies of intentions to quit smoking have been conducted in Bangladesh – a country with 22 million adult smokers [24]. This lack of evidence has hindered the development of effective policies to promote smoking cessation interventions [25]. In this study, we examined the associations between intentions to quit smoking and a range of factors, including socio-demographic characteristics, tobacco use behaviors, tobacco use attitudes, worries about health, and knowledge of the harms of tobacco smoking among a nationally representative sample of Bangladeshi adults.

## Methods

### Sample

The data for this study come from Wave 2 (March 2010 to June 2010) of the International Tobacco Control (ITC) Policy Evaluation Bangladesh Survey, a nationally representative prospective cohort survey of smokers and non-smokers aged 15 and older [26, 27]. Respondents were sampled from all six administrative divisions of Bangladesh using a stratified multi-stage probability design. Respondents were also sampled from Dhaka’s urban slums to represent the urban poor, from two tribal districts to study tobacco use in the Garo and Chakma indigenous populations, and from a land port bordering India thought to be important for cross-border tobacco trade.

In Wave 2, 3111 Wave 1 smokers were re-contacted and 622 additional smokers were recruited to replace Wave 1 respondents lost to attrition. New respondents were recruited using the same sampling design as Wave 1. Data were collected using face-to-face interviews and sampling weights were computed so that results represent the population of adult smokers. The analysis reported here is based on 2982 current smokers in Wave 2, 2337 of whom smoked cigarettes, 368 of whom smoked bidis and 277 of whom smoked both cigarettes and bidis. Complete details of the ITC Bangladesh survey are available elsewhere [28]. Ethical approval was obtained from the Office of Research Ethics at the University of Waterloo (Waterloo, Canada), and the Ethical Review Committee, Bangladesh Medical Research Council.

### Measures

#### Primary outcome measure

The primary outcome measure for this study was intentions to quit smoking. Current smokers were asked whether they were planning to quit smoking “Within the next month,” “Within the next 6 months,” or “Sometime in the future, beyond 6 months.” Smokers could also report that they were “Not planning to quit.” In this study, quit intentions were dichotomized into “any intention to quit” versus “no intention to quit”.

#### Predictor variables

The ITC Bangladesh survey measures a broad range of domains related to tobacco use, attitudes toward and perceptions of tobacco control policies. Relevant domains for this study include socio-demographic characteristics, tobacco use behaviours, tobacco use attitudes, worries about health, and knowledge of the harms of tobacco smoking.

#### Socio-demographic measures

Respondents were characterized according to their sex, age, religion (Muslim vs. non-Muslim), residence (Dhaka city, urban slums within Dhaka, areas outside Dhaka, and tribal/border areas), and marital status (married vs. not married). The ITC Bangladesh survey also assessed respondents’ highest level of formal education (illiterate, 1–8 years, 9 years or more), monthly household income (<5000 BDT, 5000–10,000 BDT, > 10,000 BDT and not reported) (IUS$ = 79BDT), and the number of smokers living in each respondent’s home.

#### Tobacco use and prior attempts to quit

Respondents were classified into three different tobacco use categories on the basis of whether they exclusively smoked cigarettes or bidis or whether they smoked cigarettes and bidis (dual use). Usual tobacco consumption was based on the self-reported number of cigarettes or bidis smoked each day for cigarette smokers and bidi smokers, respectively. For dual users, total consumption was based on the number of cigarettes and bidis smoked each day. Respondents were also asked how many of their friends or acquaintances currently smoke (0 to 5).

With respect to previous attempts to quit smoking, respondents were asked whether they had tried to quit smoking in the past year. Based on their responses, respondents were classified into those smokers who made at least one attempt vs. smokers who did not try to quit in the past year. Smokers were also asked to report the longest time they had ever been smoke-free. Responses were classified into “<1 month,” “1–6 months,” “>6 months” or “don’t know”. Finally, respondents were asked whether they had been to a doctor in the last year, and, if so, whether they had been advised to quit smoking.

#### Motivational factors

Smokers’ motivation to quit smoking was assessed by measuring (a) their opinions of cigarette and bidi smoking, (b) their expectations of future health effects if they quit smoking (outcome expectancy), (c) their level of worry about the health consequences of smoking and (d) whether they had favourable attitudes toward smoking. Opinions of cigarette smoking were classified as good or neutral vs. bad and very bad. Outcome expectancy was assessed using the question “How much do you think you would benefit from health and other gains if you were to quit smoking cigarettes permanently in the next 6 months?” Responses ranged from 1 = “not at all” to 5 = “extremely”. Concern about health was assessed by the question “How worried are you, if at all, that smoking cigarettes will damage your health in the future?” Possible responses were “not at all worried,” “a little worried,” “moderately worried” and “very worried.” Finally, respondents were asked whether they enjoy smoking too much to give it up. Responses were classified on a 5 point Likert scale from 1 = “strongly agree” to 5 = “strongly disagree.” Note that smokers could report “don’t know” to any of these items. These responses were retained in the analysis reported here and treated as an “ambivalent” category.

#### Policy relevant measures

To assess whether policy related measures might influence smokers’ intentions to quit, smokers were asked (a) how often in the last month they noticed warning labels (never, once in a while, often, very often) and (b) whether smoking is banned in indoor areas in their workplace. Responses were classified into: does not work outside the home, works outside only, no restrictions or only partial restrictions and smoking is not allowed indoors. Respondents who were employed but did not report their workplace smoking policy were retained in the analysis using the category “Employed, policy not reported”.

#### Knowledge of health effects

Finally, two composite measures assessed smokers’ knowledge of the harmful effects of (a) smoking and (b) exposure to second hand smoke. Knowledge of the harmful effects of smoking was assessed using 14 separate measures of health effects including knowledge of whether cigarette smoking causes stroke, impotence, mouth cancer, lung cancer, coronary heart disease, tuberculosis, and bronchitis and whether bidi smoking causes each of these outcomes. Affirmative responses to each item were coded with a value of 1 while all other responses were coded as 0. An overall knowledge score was created by summing each of these items. Similarly, knowledge of the harms of second-hand smoke was assessed by affirmative responses to whether second-hand smoke causes lung cancer in non-smokers, heart disease in non-smokers, asthma in children and tuberculosis. Again, an overall knowledge score was created by summing these four items.

### Statistical analysis

Unless otherwise noted, all statistical estimates and tests presented here were weighted using the Wave 2 cross-sectional sampling weight to ensure that results represent the population of adult smokers in Bangladesh. Statistical analysis, conducted using SAS version 9.4, accounted for the complex survey design. Descriptive statistics were used to estimate the percentage of smokers intending to quit smoking by important socio-demographic and tobacco use variables. Bivariate associations between quit intentions and socio-demographic, behavioural, motivational, and policy measures were assessed using the Rao-Scott *χ* ^2^ test. Differences in quit intentions by health knowledge were assessed using univariate linear regression models.

Binary logistic regression was used to estimate associations between independent predictors and intentions to quit smoking (any intention vs. no intention). All covariates described above were entered into an initial model and a final model was built using a backward selection procedure. Variables used in the construction of sampling weights (residence, sex and tobacco use), along with age, were forced into the model. In each step of the model building procedure, a sub-model was estimated by removing one covariate at a time. The Akaike information criterion (AIC) was computed for each sub-model. The sub-model having the smallest AIC statistic after removing a covariate was then selected as the best fitting sub-model. This procedure was repeated until removal of variables no longer improved model fit (i.e., no sub-model produced a smaller value for the AIC statistic). The final selected model was then re-fit using only the selected covariates in order to use as many observations as possible to predict quit intentions.

## Results

### Profile of subjects

Of the respondents (*N* = 2982), most were male (96 %), married (80 %), and Muslim (83 %); 33 % were illiterate and 54 % were aged below 40. Almost two-thirds were from areas outside Dhaka, while 22 % were from Dhaka’s urban slums (Table 1). The majority of the sample smoked cigarettes exclusively (78 %) and did not try to quit in the past year (82 %).

**Table 1:** Characteristics of the ITC Bangladesh Wave 2 sample of tobacco smokers (unweighted, *n* = 2982)

	Frequency (%)
Wave of recruitment	
1	2361 (79.2)
2	621 (20.8)
Area sample	
Dhaka (non-slum)	333 (11.2)
Dhaka slums	649 (21.8)
Areas outside Dhaka	1862 (62.4)
Tribal/border areas	138 (4.6)
Sex	
Male	2851 (95.6)
Female	131 (4.4)
Age	
15–24	511 (17.1)
35–39	1109 (37.2)
40–54	793 (26.6)
55+	569 (19.1)
Marital status	
Married	2385 (80.1)
Otherwise	592 (19.9)
Religion	
Otherwise	497 (16.7)
Muslim	2483 (83.3)
Highest level of education	
Illiterate	1002 (33.6)
1–8 years	1428 (47.9)
9+ years	550 (18.5)
Monthly household income	
< 5000 BDT	445 (14.9)
5000 to < 10,000 BDT	1398 (46.9)
10,000 BDT or more	1000 (33.5)
Not reported	139 (4.7)
Children in household	
No children in home	789 (26.5)
Only children 5 or younger	626 (21.0)
Only children aged 6 to 14	732 (24.5)
Children 14 or younger	835 (28.0)
No. household smokers	
1	1115 (37.4)
2	1306 (43.8)
3 or more	561 (18.8)
Tobacco use status	
Exclusive cigarette smoker	2337 (78.4)
Exclusive bidi smoker	368 (12.3)
Dual user	277 (9.3)
Amount smoked/day	
< = 10 sticks/day	1801 (61.9)
11–20 sticks/day	870 (29.9)
21+ sticks/day	237 (8.1)
Made a quit attempt in past year	
No attempts in past year	2383 (81.6)
At least one attempt in past year	537 (18.4)

### Patterns of intention to quit

Overall, 36 % of Bangladeshi smokers intended to quit smoking tobacco in 2010 (95 % CI: 31.9–41.2 %). With the exception of residence, quit intentions did not significantly differ by the socio-demographic characteristics of Bangladeshi smokers (Table 2). However, a smaller percentage of smokers from the tribal or border areas planned to quit smoking compared to smokers living non-tribal areas outside Dhaka (25 vs 38 %, respectively). Only 28 % of bidi smokers planned to quit, compared to 37 % of cigarette smokers and 43 % of dual users (*p* = 0.054).

**Table 2:** Percentage of adult smokers in Bangladesh having any plans to quit smoking (weighted, *n* = 2884)

	(Unwtd. Freq.)	% (95 % CI)	Rao-Scott ChiSq Test		
			*χ* ^2^	DF	*p*
Overall					
Prevalence	(901/2884)	36.4 (31.9, 41.2)			
Area sample					
Dhaka (non-slum)	(98/333)	29.3 (14.8, 49.7)	8.27	3	0.041
Dhaka slums	(101/646)	20.1 (8.1, 41.8)			
Areas outside Dhaka	(670/1769)	38.1 (33.4, 43.0)			
Tribal/border areas	(32/136)	24.7 (20.8, 29.1)			
Sex					
Male	(859/2755)	36.3 (32.0, 40.9)	0.14	1	0.713
Female	(42/129)	39.5 (22.6, 59.3)			
Age					
15–24	(157/503)	36.0 (28.2, 44.7)	4.24	3	0.237
35–39	(310/1083)	32.8 (26.7, 39.5)			
40–54	(243/764)	37.4 (30.8, 44.6)			
55+	(191/534)	41.7 (33.9, 49.9)			
Marital status					
Married	(721/2295)	36.1 (31.5, 40.9)	0.23	1	0.629
Otherwise	(177/584)	37.8 (30.3, 46.0)			
Religion					
Muslim	(753/2415)	37.3 (32.2, 42.6)	0.94	1	0.333
Otherwise	(148/467)	32.4 (24.2, 41.8)			
Highest level of education					
Illiterate	(267/945)	34.7 (28.4, 41.6)	2.38	2	0.305
1–8 years	(444/1392)	35.9 (30.6, 41.5)			
9+ years	(190/545)	41.6 (33.6, 50.0)			
Monthly household income					
< 5000 taka	(114/416)	31.3 (23.9, 39.8)	5.60	3	0.133
5000 to < 10,000 taka	(414/1343)	34.8 (29.1, 40.9)			
10,000 taka or more	(335/987)	41.6 (35.8, 47.6)			
Not reported	(38/138)	37.5 (23.7, 53.6)			
Children in household					
No children in home	(230/763)	36.9 (31.8, 42.2)	1.92	3	0.589
Only children 5 or younger	(213/602)	39.6 (33.7, 45.8)			
Only children aged 6 to 14	(207/706)	35.4 (29.3, 42.1)			
Children 14 or younger	(251/813)	34.7 (27.5, 42.7)			
No. household smokers					
1	(314/1084)	35.1 (28.9, 41.9)	2.61	2	0.271
2	(413/1253)	35.4 (30.6, 40.6)			
3 or more	(174/547)	40.5 (33.6, 47.9)			
Tobacco use status					
Exclusive cigarette smoker	(735/2336)	37.3 (31.7, 43.3)	5.84	2	0.054
Exclusive bidi smoker	(94/365)	28.1 (21.5, 35.7)			
Dual user	(72/183)	42.7 (33.3, 52.7)			

### Predictors of intention to quit

Several behavioural, motivational, policy and knowledge measures were significantly associated with intentions to quit smoking, as presented in Table 3. Table 4 presents the results of the multivariable logistic regression model developed to examine correlates of quit intentions after adjusting for other covariates. Not all covariates presented in Table 3 were chosen by the selection procedure for inclusion in the final logistic regression model. However, all relationships between covariates and quit intentions in the multivariable model were consistent with the bivariate relationships presented in Table 3. With respect to behavioural variables, smokers smoking fewer cigarettes per day had significantly higher odds of planning to quit compared to smokers smoking 21 or more cigarettes per day. Similarly, smokers who ever tried to quit had 2.8 times higher odds of planning to quit compared to smokers who had never tried to quit. Receiving advice to quit from a doctor also influence the odds of planning to quit smoking (OR = 1.86, compared to smokers who did not visit a doctor in the past 6 months).

**Table 3:** Smoking behaviours associated with intentions to quit among all tobacco smokers (weighted, *n* = 2884)

	Unwtd. Freq.	% (95 % CI)	Rao-Scott ChiSq Test		
			*χ* ^2^	DF	*p*
Number of friends/acquaintances who smoke					
No/only 1 friend smokes	(21/53)	34.6 (13.8, 63.8)	19.32	4	<0.001
2 friends smoke	(90/195)	52.1 (42.3, 61.8)			
3 friends smoke	(187/521)	37.4 (29.8, 45.7)			
4 friends smoke	(213/751)	28.6 (23.4, 34.4)			
5 friends smoke	(379/1242)	31.4 (25.5, 37.9)			
Amount smoked/day					
< = 10 sticks/day	(605/1784)	36.0 (28.9, 43.7)	6.64	2	0.036
11–20 sticks/day	(220/804)	28.8 (22.7, 35.6)			
21+ sticks/day	(59/224)	25.4 (19.3, 32.6)			
Made a quit attempt in past year					
No attempts in past year	(573/2296)	26.4 (21.0, 32.5)	29.14	1	<0.001
At least one attempt in past year	(322/528)	60.1 (47.8, 71.3)			
Longest duration smoke-free					
Never quit	(380/1664)	24.1 (18.7, 30.5)	82.61	4	<0.001
< 1 month	(297/685)	44.8 (38.6, 51.1)			
1 to 6 months	(178/340)	53.2 (41.5, 64.6)			
> 6 months	(11/38)	21.0 (9.7, 39.7)			
Don’t know	(28/97)	27.5 (16.9, 41.4)			
Enjoy smoking					
Ambivalent	(24/373)	6.3 (3.3, 11.7)	213.28	3	<0.001
Agree/strongly agree	(570/1744)	33.1 (27.9, 38.7)			
Neither	(45/176)	28.4 (20.4, 38.0)			
Disagree/strongly disagree	(262/591)	46.6 (39.9, 53.4)			
Bad opinion of cigarette smoking					
Positive/neutral	(5/36)	10.2 (2.9, 30.0)	4.96	1	0.026
Bad/very bad	(895/2824)	32.8 (27.3, 38.8)			
Bad opinion of bidi smoking					
Positive/neutral	(6/38)	10.0 (2.6, 31.3)	4.50	1	0.034
Bad/very bad	(894/2828)	32.8 (27.3, 38.8)			
Bad opinion of hookah smoking					
Positive/neutral	(24/109)	24.9 (15.5, 37.5)	1.29	1	0.255
Bad/very bad	(859/2688)	33.1 (27.2, 39.5)			
Health benefits if quit smoking					
Ambivalent	(6/111)	2.3 (0.4, 7.1)	258.33	3	<0.001
Little benefit	(275/1226)	23.9 (18.3, 30.4)			
Moderate benefit	(37/272)	16.9 (10.8, 25.4)			
Great benefit	(582/1263)	45.5 (40.2, 50.9)			
Worry about future health					
Ambivalent	(9/184)	5.1 (2.3, 11.2)	111.32	4	<0.001
Not at all worried	(20/170)	11.1 (6.3, 19.0)			
A little worried	(260/898)	30.3 (25.8, 35.3)			
Moderately worried	(432/1231)	35.2 (28.1, 43.1)			
Very worried	(178/389)	50.2 (41.7, 58.6)			
Noticed warning labels					
Unaware of labels	(79/424)	21.3 (16.1, 27.5)	16.57	2	<0.001
Never/once in a while	(325/937)	36.6 (30.0, 43.8)			
Often/whenever smoke	(488/1439)	34.2 (27.8, 41.1)			
Workplace smoking policy					
Employed, policy not reported	(57/159)	38.9 (26.5, 52.9)	32.13	4	<0.001
Not employed outside home	(440/1148)	39.8 (33.5, 46.4)			
Employed, does not work indoors	(255/1112)	24.5 (19.2, 30.8)			
No/partial restrictions only	(71/244)	30.9 (22.8, 40.3)			
Smoking not allowed indoors	(77/211)	31.6 (21.7, 43.7)			
Received advice from doctor to quit smoking					
Did not visit doctor	(627/2128)	30.1 (25.0, 35.9)	33.74	2	<0.001
Visited, no advice to quit	(90/392)	24.8 (17.9, 33.2)			
Visited & advised to quit	(183/363)	51.4 (40.6, 62.0)			
Knowledge of smoking harms^a^					
Low	(135/821)	20.5 (11.7, 33.4)	28.26	2	<0.001
Moderate	(177/619)	31.0 (25.9, 36.7)			
High	(589/1443)	48.3 (43.8, 52.8)			
Knowledge of effects of SHS^a^					
Low	(155/869)	19.0 (11.5, 29.9)	38.29	2	<0.001
Moderate	(124/432)	36.0 (28.0, 44.8)			
High	(900/2870)	48.5 (44.5, 52.5)			

^a^Knowledge indices were ranked into approximate tertiles to explore the relationship between knowledge and quit intentions

**Table 4:** Predictors^a^ of intentions to quit (any intention vs no intention) among all tobacco smokers (*n* = 2637)

	OR (95 % CI)	*χ* ^2^	DF	*p* value
Sampling area				
Dhaka non-slum areas vs. Dhaka slums	1.34 (0.43, 4.20)	23.83	3	<0.001
Areas outside Dhaka vs. Dhaka slums	2.47 (1.08, 5.63)			
Tribal & border areas vs. Dhaka slums	0.69 (0.29, 1.66)			
Sex				
Women vs. men	1.78 (0.77, 4.10)	1.84	1	0.175
Age group				
25–39 vs. 15–24	0.84 (0.59, 1.18)	4.65	3	0.199
40–54 vs. 15–24	0.96 (0.62, 1.48)			
55+ vs. 15–24	1.10 (0.80, 1.51)			
Type of smoker				
Cigarette vs. dual user	0.75 (0.47, 1.18)	1.65	2	0.437
Bidi vs. dual user	0.83 (0.57, 1.20)			
Children at home				
Only children 5 or younger vs. no children	1.62 (1.24, 2.12)	19.71	3	<0.001
Only children 6–14 vs. no children	1.00 (0.77, 1.28)			
Children in both age groups vs. no children	0.82 (0.61, 1.08)			
Amount smoked per day (cigarettes and/or bidis)				
10 or fewer vs. 21+ sticks/day	2.08 (1.34, 3.22)	13.93	2	<0.001
11–20 vs. 21+ sticks/day	1.59 (0.87, 2.90)			
Made a quit attempt in past year				
At least one attempt vs none	2.79 (1.70, 4.56)	16.68	1	<0.001
Enjoys smoking				
Does not enjoy smoking vs. enjoys smoking	1.45 (1.00, 2.08)	47.24	3	<0.001
Neither enjoys/not enjoy vs. enjoys smoking	1.08 (0.73, 1.61)			
Ambivalent vs. enjoys smoking	0.18 (0.09, 0.36)			
Perceptions about the benefit of quitting				
Great benefit if quit vs. little benefit	2.10 (1.63, 2.72)	83.32	3	<0.001
Moderate benefit if quit vs. little benefit	0.71 (0.48, 1.06)			
Ambivalent vs. little benefit	0.01 (0.00, 0.08)			
Worry about the health consequences of smoking				
Very worried about health effects vs. Not at all	8.66 (3.89, 19.28)	44.66	4	<0.001
Moderately worried vs. Not at all	4.28 (1.88, 9.74)			
A little worried vs. Not at all	3.95 (1.82, 8.59)			
Ambivalent vs. Not at all	0.95 (0.36, 2.50)			
Noticed warning labels on tobacco packaging				
Often/whenever smoke vs. unaware of warning labels	0.67 (0.44, 1.04)	7.06	2	0.029
Never/rarely vs. unaware of warning labels	0.97 (0.63, 1.50)			
Workplace smoking policy				
Not allowed indoors vs. does not work indoors	1.96 (1.32, 2.91)	22.77	4	<0.001
No/partial bans vs. does not work indoors	1.73 (1.15, 2.61)			
Does not work outside home vs. does not work indoors	1.47 (1.11, 1.95)			
Employed, policy not reported vs. does not work indoors	1.97 (1.21, 3.19)			
Visited doctor in last year				
Visited & received advice vs. did not visit	1.86 (1.31, 2.63)	18.42	2	<0.001
Visited, no advice vs. did not visit	0.50 (0.25, 0.98)			
Knowledge about the harms of smoking				
1 point increase	1.08 (0.99, 1.18)	2.75	1	0.097
Knowledge about the harms of second-hand smoke				
1 point increase	1.20 (1.08, 1.33)	12.55	1	<0.001

^a^Odds ratios were estimated from a weighted multivariable logistic regression model that accounts for the complex sampling design. Reported odds ratios are therefore adjusted odds ratios that control for all other covariates included in the model

Motivational factors influence the odds of planning to quit. Smokers who believed there was great benefit in quitting had 2.1 times higher odds of planning to quit compared to smokers who saw little benefit in quitting. Enjoyment of smoking also predicted the odds of planning to quit: smokers who do not enjoy smoking had 1.4 times higher odds of planning to quit compared to smokers who enjoy smoking. Smokers’ concern about the health consequences significantly influenced their odds of planning to quit: smokers who were very worried about their health had 8.7 times higher odds of planning to quit compared to smokers who were not at all worried. Even small amounts of concern were associated with higher odds of planning to quit such that smokers who were moderately worried had 4.2 times higher odds of planning to quit and smokers who were only a little worried about health had 3.9 times the odds of planning to quit.

Tobacco control policies may influence smokers’ intentions to quit. Specifically, smokers employed in indoor areas where smoking is banned had 2.0 times higher odds of planing to quit compared to smokers who worked outdoors. Even partial bans were associated with intentions to quit (OR = 1.73). Furthermore, knowledge of the harmful effects of second-hand smoke was associated with increased odds of planning to quit. Similarly, smokers living in homes with children aged 5 or younger had significantly higher odds of planning to quit compared to smokers having no children in the home (OR = 1.62). However, awareness of the harmful effects of smoking on one’s own health was not associated with intentions to quit (*p* = 0.09).

Additional factors not included in the final multivariable model may influence smokers’ intentions to quit. The percentage of smokers who planned to quit was lower among smokers having a greater number of friends who smoke: 31 % of smokers with five smoking friends planned to quit compared to 52 % of smokers having only two smoking friends (Table 3). Likewise, only 24 % of smokers who never tried to quit planned to quit compared to 53 % of smokers who had previously quit for one to six months. Approximately one-third of smokers having negative opinions of cigarette, bidi, or hookah smoking planned to quit while smokers having positive opinions of tobacco smoking were less likely to plan to quit.

## Discussions

This is the first nationally representative survey to study predictors of intentions to quit smoking among Bangladeshi adults. The findings demonstrate that interest in quitting among adult Bangladeshi smokers is low (36 %) compared to the rates in developed regions or countries. For example, 52 % of Hong Kong Chinese plan to quit [29], and rates in many developed countries of the West are even higher, ranging from 65 to 81 % [30]. Among Korean adults, 75 % of adult smokers plan to quit [22]. However, only 15 % of Chinese adult smokers plan to quit (range 15 to 31 %) [23]. Even though the percentage of smokers planning to quit in our study was higher than the reported rates among Chinese smokers [23], the percentage is low. This low quitting intention among Bangladeshi smokers is due to several factors, including lack of public health campaigns that focus on the benefits and harms of quitting smoking, lack of smoking cessation programs (e.g., quitline or clinic) that are available in Bangladesh, and the lack of available smoking cessation medications in Bangladesh. The low rate of quitting intention among the Bangladeshi adults underscore the need for comprehensive policy initiatives that would encourage smokers to think about quitting. At the same time, the national tobacco control program, should incorporate smoking cessation within its service framework.

Many of the predictors of quit intentions identified in this study are similar to those identified in previous studies with some differences. Contradicting the findings of previous studies [8–10], demographic factors such as age, gender, income, education were not predictors of intention to quit smoking in the current study. The lack of demographic differences in quit intentions might be related to the socio-cultural differences that might encourage smoking and quitting. However, the relation between demographic characteristics and intentions to quit are not always consistent [23, 31].

Previous studies have noted that nicotine dependence predicts smoking cessation and quit intentions [32–34]. In the current study, smoking fewer cigarettes per day, a proxy measure of nicotine dependence, was an independent predictor of quit intentions. This suggests that physical dependence could affect one’s desire to quit smoking and underscores the need to tailor smoking cessation strategies based on the smokers’ level of nicotine dependence [35, 36].

The relationship between smoking restrictions and intention to quit is not well studied. In a study of Korean adults [22], intention to quit was associated with home smoking restrictions but not with workplace smoking restrictions. In another study, both household and workplace smoking restrictions were associated with intention to quit and successful cessation [37]. In our study, workplace smoking policy predicted intentions to quit. This finding reinforces the fact that smoking bans may increase smokers’ motivation to think about quitting and encourage them to attempt to quit [37] thereby promoting smoking cessation. Any policy implementation should be assesses periodically for reinforcement, because the impact of a new policy on smokers’ intentions to quit may peak initially and then subside as time passes since the policy was implemented [33, 38].

The finding that past quitting efforts (i.e. tried to quit in the past) were associated with quit intentions compared to making no effort (i.e. never tried), is of note. These findings are likely due to differences in smokers’ readiness to quit, because past quitters who are already motivated may try again while smokers without prior attempts may simply not be ready to quit and therefore have no intention to quit. This suggests the need to motivate smokers to think about quitting by raising awareness of the benefits of quitting smoking. This should also encourage smokers to make frequent quit attempts, regardless of perceived likelihood of success on that quit attempt. In a study of Chinese adult smokers by Feng et al. [23], past quitting experience predicted subsequent quitting attempts. In this study perceptions about the benefit of quitting was strongly associated with intention to quit smoking, underscoring the need for policies that would focus on improving the perception of benefits of quitting smoking, probably through the educational campaign [4, 29].

The findings of the independent effect of motivational variables (i.e. worry about the harms of smoking, perception about benefits of quitting, enjoying smoking) on intentions to quit smoking in the current study is consistent with earlier studies among Chinese [23], Korean [22] and the Dutch smokers [38]. Increased knowledge about the harms of second hand smoke also predicted quit intentions in the current study reflecting the need to maintain adequate knowledge level among the public. Educational campaigns should focus on increasing public knowledge and awareness about the harms of tobacco and promote the benefits of smoking cessation.

We found that having a young child at home predicted quit intentions among Bangladeshi adults. This might be due to their increased knowledge about the harms of second-hand smoke (SHS) exposure as was found in the current study and their concern about childhood SHS exposure [26]. In our earlier study using ITC Wave 1 data, 89 % of parents appropriately knew that SHS exposure may cause asthma in children [26]. The same study also reported that being concerned about a child’s SHS exposure was a predictor of adopting a smoke-free policy in the home. Tobacco control strategies should continue with awareness campaigns to maintain high levels of knowledge about smoking and SHS, and to raise awareness of smoking and SHS exposure among the public. Utilizing child’s health as a motivating factor, engaging pediatricians to encourage smoking parents to think about quitting and making quit attempt is effective [39], which would benefit both smokers and their children. Our findings showed that visiting a doctor and receiving advice predicted smokers’ intention to quit. This reinforces the fact that doctor’s advice, even if it is only brief advice, is effective in promoting smoking cessation [40, 41]. Physicians should be encouraged to routinely identify whether their patients smoke and provide them with brief advice to quit or to at least make an attempt to quit [42].

The current study has several limitations. First, our analysis was limited to daily smokers, so the findings may not be applicable to non-daily smokers. However, according to Wave 1 data, 96 % of cigarette smokers/dual users smoke cigarettes daily and 94 % of dual users smoke bidis daily. Thus, our results represent the vast majority of smokers in Bangladesh. Second, nicotine dependence has been reported as a predictor of smoking cessation and quitting intentions in previous studies [32–34]. However, we were unable to include nicotine dependence as a predictor of intentions due to the way the data were collected in Wave 1. We tried to compensate for this by using the total number of cigarettes smoked per day as a proxy measure of dependence.

## Conclusions

The present study identified several predictors of intention to quit among Bangladeshi smokers, including past quitting experience, the number of cigarettes smoked daily, having a young child (aged 5 or younger) at home, health concerns, health services utilization and motivational factors. These predictors are fairly similar to those found among smokers in other developed countries. Population based tobacco control programs and policies should consider these predictors in the design of interventions to increase quitting among smokers in Bangladesh. At the same time, measures are necessary to increase intentions to quit among current smokers, because intention to quit is the first step towards the goal of quitting smoking completely. These findings has implications for Bangladesh and other developing countries, especially those in the Southeast Asia region, to strengthen the current tobacco control policies by incorporating smoking cessation within the policy agenda while promoting smoking ban in public places, workplaces and home.

## Abbreviations

AIC: Akaike information criterion
BDT: Bangladeshi Taka
ITC: International Tobacco Control Policy Evaluation Project
SHS: Second-hand smoke
WHO: World Health Organization
